# High Glucose Aggravates the Detrimental Effects of Pancreatic Stellate Cells on Beta-Cell Function

**DOI:** 10.1155/2014/165612

**Published:** 2014-07-03

**Authors:** Min Zha, Wei Xu, Qing Zhai, Fengfei Li, Bijun Chen, Zilin Sun

**Affiliations:** ^1^Department of Endocrinology, Zhongda Hospital, Institute of Diabetes, Medical School, Southeast University, Nanjing, Jiangsu 210009, China; ^2^Department of Endocrinology and Genetic Metabolism, Yijishan Hospital of Wannan Medical College, Wuhu, Anhui 241001, China

## Abstract

*Background and Aims*. We here assess the effects of PSCs on *β*-cell function and apoptosis* in vivo* and* in vitro*.* Materials and Methods.* PSCs were transplanted into Wistar and Goto-Kakizaki (GK) rats. Sixteen weeks after transplantation, *β*-cell function, apoptosis, and islet fibrosis were assessed.* In vitro* the effects of PSCs conditioned medium (PSCs-CM) and/or high concentration of glucose on INS-1 cell function was assessed by measuring insulin secretion, INS-1 cell survival, apoptosis, and endoplasmic reticulum stress (ER stress) associated CHOP expression.* Results*. PSCs transplantation exacerbated the impaired *β*-cell function in GK rats, but had no significant effects in Wistar rats.* In vitro*, PSCs-CM caused impaired INS-1 cell viability and insulin secretion and increased apoptosis, which were more pronounced in the presence of high glucose. *Conclusion.* Our study demonstrates that PSCs induce *β*-cell failure* in vitro* and* in vivo*.

## 1. Introduction

Type 2 diabetes mellitus (T2DM) is a clinical syndrome characterized by elevated blood glucose caused by a combination of insulin resistance and progressive failure of insulin secretion by the *β*-cells in pancreatic islets of Langerhans [1, 2]. The cellular mechanisms underlying *β*-cell failure in T2DM are not well understood, but several recent studies suggest that pancreatic stellate cells (PSCs) might play an important role in this process [3–7]. Thus, PSCs have a well-established role in the pathological fibrotic responses observed in inflammatory pancreatic disorders such as chronic pancreatitis and pancreatic cancer [8–10], and islet fibrosis has been reported in the late stage of T2DM in humans and in animal models [2, 3, 11–15]. PSCs are activated to proliferate and generate fibrotic extracellular matrix (ECM) by a range of environmental stimuli which are also associated with T2DM, including hyperglycemia [3, 16], renin-angiotensin system (RAS) activation [17], oxidative stress [4, 13], platelet-derived growth factors [18], and inflammatory cytokines [19]. Studies in animal models of T2DM have shown that treatments which improve glucose tolerance—including angiotensin-converting enzyme inhibitors (ACEI), antioxidants, conophylline, and inhibition of NADPH oxidase, also suppress PSC activation, reduce islet fibrosis, and improve *β*-cell mass and function [3, 5, 7, 20]. More recently, PSCs were shown to induce apoptosis in a *β*-cell line* in vitro*, although functional correlates were not reported in this study [9].

Together, these observations are consistent with an important role for PSCs in the progressive *β*-cell dysfunction associated with the development of T2DM. In this study we have combined* in vivo* and* in vitro* models to explore the direct effects of PSCs on *β*-cell function and to investigate the importance of the diabetic microenvironment in PSC/*β*-cell interactions.

## 2. Materials and Methods

### 2.1. Experimental Animals

Wistar and GK rats were housed three per cage and given access to food and water ad libitum. After a one-week acclimation period, the rats were used for experiments.

### 2.2. Isolation of PSCs

PSCs were isolated from 8-week-old Wistar rats as described previously [21]. The PSCs were cultured in Dulbecco's modified Eagle's medium/Ham's F12 (DMEM/F12, Gibco, CA, USA) containing 10% fetal bovine serum (FBS, Gibco, CA, USA).

### 2.3. Intrapancreatic Transplantation of PSCs

PSCs were used for transplantation at passages 4-5. Three experimental groups were established, each of at least five experimental animals: (1) untreated GK (GU) and Wistar (WU) rats; (2) sham-operated GK (GS) and Wistar (WS) rats; and (3) PSC-transplanted GK (GT) and Wistar (WT) rats. Intrapancreatic PSC transplantation was performed as described [22, 23]. 1 × 10^6^ PSCs suspended in 100 *μ*L PBS were directly injected into the pancreas of transplanted groups. Sham-operated controls received multiple intrapancreatic injections of PBS alone. Sixteen weeks after transplantation, *β*-cell function and islet morphometric analysis were detected.

### 2.4. Measurement of Blood Glucose, Insulin, and HbA1c

Blood glucose and insulin levels were measured before and 30, 60, 90, and 120 min after glucose administration. Insulin secretion was measured using a rat insulin ELISA kit (Millipore, MA, USA). The area under the curve for glucose (AUC_g_) and insulin (AUC_i_) was calculated using a trapezoidal estimation from the values. HbA1c was measured using the DCA 2000+ HbA1c reagent kit (Bayer, Berlin, Germany) [24].

### 2.5. Immunohistochemical and Morphometric Analysis of Pancreas

To measure the *β*-cell mass, apoptosis, and islet fibrosis, insulin immunofluorescent staining, TUNEL (Roche, Upper Bavaria, Germany), and Masson's trichrome stain (Sigma, Victoria, Australia) were performed on sections of pancreas, respectively. The morphometric analysis was done manually using Image J software to measure the insulin-positive area, the fibrosis-positive area, and the islet and the total pancreatic area at 200× magnification. The relative *β*-cell mass was expressed as the insulin-positive area/total pancreas area. The islet fibrosis was calculated as the fibrosis-positive area/islet area. To measure *β*-cell apoptosis, TUNEL-positive/insulin-positive cells were calculated.

### 2.6. Preparation of Conditioned Medium from PSC (PSCs-CM)

To prepare PSCs-CM, cells grown to near confluence were cultured for 48 h in DMEM/F12 serum-free medium supplemented with 0.2% BSA (5 × 10^6^ cells/25 mL medium). The medium was collected, centrifuged to remove any nonadherent cells, and stored at −20°C until use.

### 2.7. Culture and Experimental Treatments of INS-1 Cells

The rat-derived, insulin-secreting INS-1 cell line was treated with PSCs-CM diluted 50% (v/v) with standard RPMI-1640 (CM group). To mimic the hyperglycemic microenvironment in the GK rat, other groups of INS-1 cells were treated by exposure to high glucose alone (25 mM, HG group) or to a combination of high glucose and PSC-conditioned medium (CM + HG group). Cells were treated for 24 or 48 h, after which insulin secretion, cell viability, apoptosis, and ER-stress were assessed.

### 2.8. Cell Viability Assay

The Cell Counting Kit-8 (CCK-8, sigma, CA, USA) was used for quantitation of viable cell numbers in cytotoxicity assays. After 24 or 48 h treatment, the viable cells were evaluated by absorbance measurements at 450 nm (*A*_450 nm_) using an automicroplate reader.

### 2.9. Flow Cytometry Analysis of Apoptotic Cells

Apoptosis was assessed by flow cytometry using the Annexin V-FITC/propidium iodide (Annexin V/PI) apoptosis detection kit (Promega, WI, USA) according to the manufacturer's protocol. After treatment for 24 h, approximately 1 × 10^5^ INS-1 cells were used for each analysis, and experiment was repeated on three separate occasions.

### 2.10. Quantitative Real-Time PCR and Western Blot Analysis of C/EBP Homologous Protein (CHOP)

The expression by INS-1 cells of CHOP, an established marker for ER-stress, was assessed at protein and mRNA levels using immunoblotting and real-time quantitative RT-PCR, respectively. As a positive control, INS-1 cells were treated with thapsigargin (1 *μ*mol/L, 24 h), an established ER stress-inducing agent.

### 2.11. Insulin Secretion

Measurements of potassium-stimulated insulin secretion (KSIS) by INS-1 cells were performed as described [25]. Insulin secretion and insulin content in cell lysates were measured using an insulin radioimmunoassay kit (Beijing Technology Company, Beijing, China).

### 2.12. Statistical Analysis

Data were presented as the means ± SE. Statistical significance was determined by unpaired Student's *t*-test or one-way variance (ANOVA), as appropriate, and differences between treatments were considered statistically significant at *P* < 0.05. All of the statistical analyses were performed using the Statistical Product and Services Solutions (SPSS) package.

## 3. Results

### 3.1. Transplanted PSCs Impair Glucose Tolerance and *β*-Cell Function* In Vivo*

**Figure 1:**
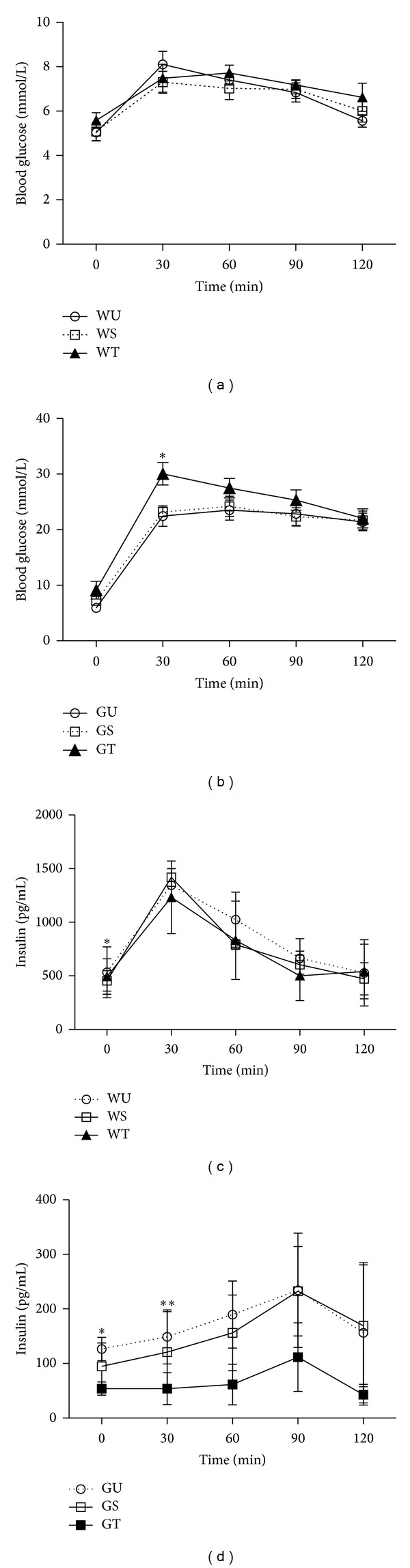
Transplanted PSCs impair glucose tolerance and *β*-cell function* in vivo*. PSC-treatment had no effects on glucose clearance after oral glucose loading (a) or on glucose-stimulated insulin secretion (c) in Wistar rats. However, in contrast, PSC transplantation exacerbated the impaired glucose tolerance of GK rats (b), and the reduced levels of insulin secretion observed in control GK rats (d) were further suppressed by intrapancreatic transplantation of PSCs. All data were expressed as mean ± SE (*n* = 5). *P < 0.05, **P < 0.01 versus untreated group.

To determine whether the PSCs have direct effects on *β*-cell* in vivo*, we assessed the effects of intrapancreatic transplantation of PSCs in Wistar and GK rats. PSCs treatment had no effect on glucose tolerance in Wistar rats, as shown in Figure 1(a). Thus, there was no significant difference in glucose clearance after oral glucose loading between the three Wistar treatment groups (WU, WS, and WT). In contrast, PSC transplantation exacerbated the impaired glucose tolerance of GK rats.  Figure 1(b) shows that all of the GK groups have impaired glucose tolerance (Figures 1(a) and 1(b)). Moreover, compared with GU and GS, GT group had significantly elevated blood glucose at 30 min in the GT group (*P* < 0.05, Figure 1(b)), leading to a significantly increased AUC_g_ (2953.2 ± 296.1 versus 2474.4 ± 374 and 2525.4 ± 273.3 mmol/L*·*min, *P* < 0.05).

In accordance with the glucose tolerance curves, PSC-treatment had no effects on glucose-stimulated insulin secretion in Wistar rats (Figure 1(c)). The reduced levels of insulin secretion observed in control GK rats (GU, GS; Figure 1(d)) were further suppressed by intra-pancreatic transplantation of PSCs, with the GT groups showing significantly decreased plasma insulin at 0 and 30 minutes after oral glucose, resulting in significantly reduced AUC_i_ in the GT group compared to the GU and GS groups (8259.2 ± 2345.8 versus 21364.9 ± 8173.4 and 19245.7 ± 4191.9 pg/mL*·*min, *P* < 0.05).

Measurements of HbA1c confirmed the chronic hyperglycemic/diabetic status of the GK rats, with significantly higher HbA1c levels in all GK treatments groups compared to normal Wistar rats. Intrapancreatic transplantation of PSCs had no effect on HbA1c levels in Wistar rats (WT), but caused significant elevations in GK rats (9.8 ± 1.4% versus 8.8 ± 1.0% and 8.5 ± 1.0%, *P* < 0.05).

### 3.2. Transplanted PSCs Reduce *β*-Cell Mass and Increase Cell Apoptosis* In Vivo*

**Figure 2:**
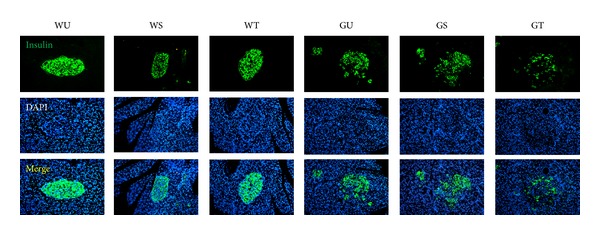
Transplanted PSCs reduce *β*-cell mass* in vivo*. Insulin immunofluorescent staining was performed on pancreatic sections. Representative images of insulin immunofluorescence staining (green) and nuclei labeled by DAPI (blue) were shown. All images were taken at the same magnification (×200).

To determine whether the* in vivo* effects of PSCs to impair glucose tolerance in GK rats were caused by direct effects on *β*-cells, we used immunofluorescence methods to assess *β*-cell mass and apoptosis in the pancreas* in situ*. PSC transplantation in GK rats (GT) caused a large reduction in the *β*-cell mass (~40%, *P* < 0.05, Figure 2), and a significant increase in *β*-cell apoptosis (~2-fold, *P* < 0.05) when compared to untreated or sham-operated controls (GU, GS). In contrast, PSC transplantation had no detectable effects on *β*-cell mass or apoptosis in Wistar rats, in accordance with the lack of effects on glucose tolerance and HbA1c in these normoglycemic control rats.

### 3.3. Transplanted PSCs Increase Islet Fibrosis* In Vivo*

**Figure 3:**
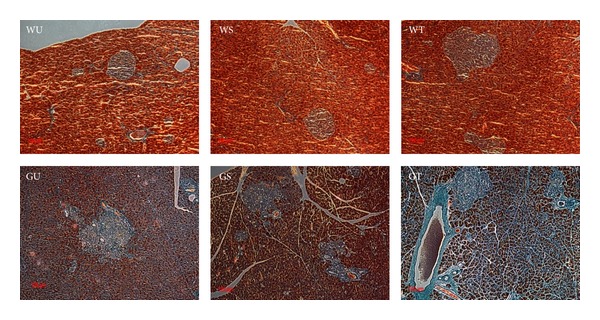
Intrapancreatic PSCs increase islet fibrosis* in vivo*. Masson's trichrome staining was performed to show the islet fibrosis. The degree of islet fibrosis in GK rats was higher than that in normal Wistar rats, and the extent of islet fibrosis in GK rats was significantly increased after intrapancreatic transplantation of PSCs. All images were taken at the same magnification (×200).

Total collagen deposition evaluated with Masson's trichrome stain showed extensive staining in the wall of blood vessels and pancreatic ducts, as well as in fibrotic islets, as shown in Figure 3. The degree of islet fibrosis in GK rats was higher than that in normal Wistar rats, and the extent of islet fibrosis in GK rats was significantly increased (~1.3-fold, *P* < 0.05) after intrapancreatic transplantation of PSCs (GT) when compared to untreated (GU) or sham-operated (GS) GK rat islets.

### 3.4. PSCs-CM Impairs INS-1 Cell Viability and Function* In Vitro*

**Figure 4:**
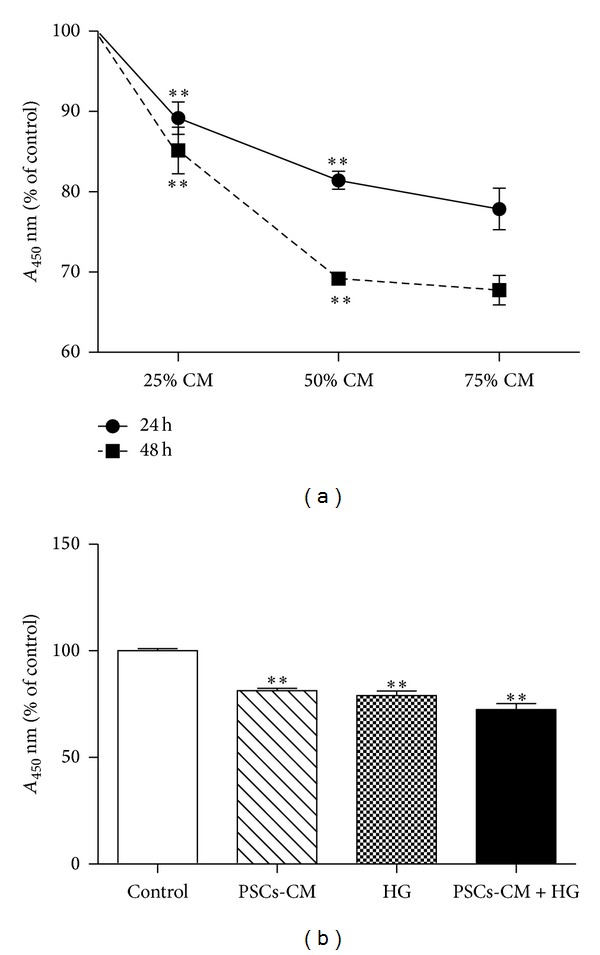
PSCs-CM impairs INS-1 cell viability* in vitro.* PSCs-CM was supplemented into the medium, forming a concentration ratio of: 0, 25, 50 and 75%. Incubation of INS-1 cells with PSCs-CM at 25, 50 and 75% (v/v) caused a significant and concentration dependent reduction in cell number compared to control INS-1 cells incubated in standard tissue culture medium. The deleterious effects of PSCs-CM were significant by 24 h, and were further enhanced after 48 h treatment (a). (b) shows that the treatment with PSCs-CM or HG for 24 h were alone sufficient to reduce INS-1 cell viability, and that their effects were additive, with the combination of PSCs-CM and HG exerting a significantly more detrimental effect than either treatment alone. All data were expressed as mean ± SE (*n* = 9), *P < 0.05, **P < 0.01 versus control.

Treatment of INS-1 cells with PSCs-CM for 24 or 48 h* in vitro* had marked effects on INS-1 cell viability and function.  Figure 4(a) shows the decline in INS-1 cells viability determined using the CCK-8 cell viability assay, in which *A*_450 nm_ is proportional to viable cell number. Incubation with PSCs-CM at 25%, 50%, and 75% (v/v) caused a significant and concentration-dependent reduction in cell number compared to control INS-1 cells incubated in standard tissue culture medium. The deleterious effects of PSCs-CM were significant by 24 h and were further enhanced after 48 h treatment (Figure 4(a)). To investigate* in vitro* interactions between PSCs and high concentrations of extracellular glucose, INS-1 cells were incubated for 24 h with 50% PSCs-CM in the presence or absence of 25 mM glucose (HG).  Figure 4(b) shows that both PSCs-CM and HG were alone sufficient to reduce INS-1 cell viability and that their effects were additive, with the combination of PSCs-CM and HG exerting a significantly more detrimental effect than either treatment alone (Figure 4(b), *P* < 0.01).

**Figure 5:**
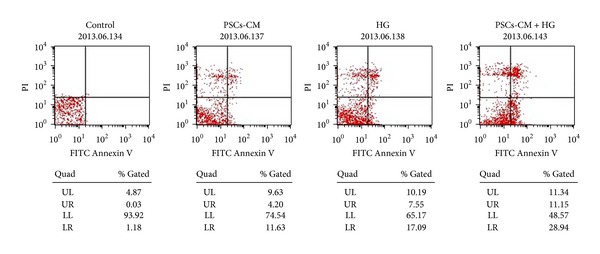
PSCs-CM increases INS-1 cell apoptosis* in vitro.* The apoptosis of INS-1 cells was detected by Annexin V-FITC/PI apoptosis detection kit. Both PSCs-CM and HG alone caused marked and significant increases in INS-1 cell apoptosis, and the effects of both treatments on apoptosis were additive, with the combination of PSCs-CM and HG exerting a significantly more detrimental effect than either treatment alone.

The PSCs-CM and/or HG-dependent reductions in INS-1 cell viability were associated with increased apoptosis, as measured using the Annexin V-FITC/PI apoptosis detection kit, with Annexin V^+^/PI^−^ cells being considered as in the early stages of apoptosis. In accordance with their effects on cell viability, both PSCs-CM and HG alone caused marked and significant increases in INS-1 cell apoptosis, as shown in Figure 5. As for INS-1 cell viability, the effects of both treatments on apoptosis were additive, with the combination of PSCs-CM and HG exerting a significantly more detrimental effect than either treatment alone (6.1 ± 0.7-, 7.9 ± 0.3-, and 16.3 ± 0.5-fold of control).

**Figure 6:**
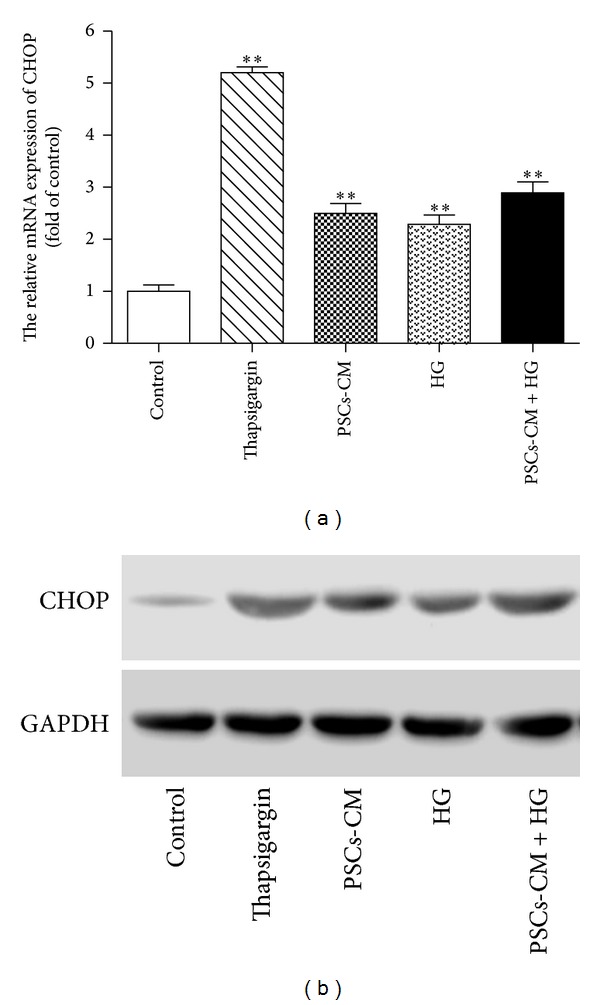
PSCs-CM induces ER-stress in INS-1 cells* in vitro*. CHOP mRNA (a) and protein (b) expression were up-regulated in INS-1 cells exposed for 24 h to PSCs-CM or HG alone and, again, their effects were additive.

Measurement of CHOP expression suggests that the effects of PSCs-CM and HG on INS-1 cell viability and apoptosis may be mediated through ER stress, at least in part, as shown in Figure 6. CHOP mRNA expression was upregulated in INS-1 cells exposed for 24 h to PSCs-CM or HG alone and, again, their effects were additive (Figure 6(a)). The increases in CHOP mRNA were associated with increased levels of CHOP protein expression, as shown in Figure 6(b). As expected, treatment with thapsigargin induced a marked increase in both CHOP mRNA and protein, indicated by the significantly upregulated expression of CHOP and validating CHOP expression as a marker for ER stress.

The deleterious effects of PSCs on INS-1 cell viability were also associated with reduced insulin secretory function. Thus, KSIS by INS-1 cells was significantly reduced by treatment (24 h) with both PSCs-CM (71.3 ± 20.3% of control, *P* < 0.05) and HG (65.8 ± 24.6% of control, *P* < 0.01) alone, with the combination of PSCs-CM and HG exerting a significantly more inhibitory effect on insulin secretion than either treatment alone (37.3 ± 16.9% of control, *P* < 0.01).

## 4. Discussion

The involvement of PSCs in pancreatic fibrosis in response to chronic inflammation or pancreatic cancer is well established [8–10, 26–30], but there have been many fewer studies into their potential role in islet fibrosis and progressive *β*-cell failure in T2DM. For the first time, PSCs transplantation and PSCs-CM were used to detect the direct effects of PSCs on *β*-cells.

Previous studies have linked activation of endogenous PSCs with the development of fibrosis/T2DM [3, 6, 7, 20], and in the current study we have used* in vivo* and* in vitro* models to demonstrate a direct deleterious effect of PSCs on *β*-cell function, leading to *β*-cell loss and impaired insulin secretion and glucose tolerance. These observations are consistent with a pivotal role for PSCs in the pathogenesis of T2DM.

Our* in vivo* experiments using intrapancreatic transplantation of PSCs demonstrate that PSCs did not influence *β*-cell mass, insulin secretion, glucose handling, or HbA1c in normoglycemic Wistar rats, but had marked effects to exacerbate all of these (patho)physiological parameters in GK rats, which are an established model of spontaneous-onset T2DM [2, 31]. Before birth, GK rats present decreased *β*-cell mass and low plasma insulin as compared with Wistar control rats, proceeding to hyperglycemia and insulin resistance around weaning (3-4 weeks of age) [2]. The development of T2DM in the GK rat is associated with chronic hyperglycemia [31, 32], activation of the renin-angiotensin system (RAS), and increased levels of oxidative stress [33–35] and inflammatory cytokines [2, 31, 32], all of which are known to have marked effects to activate PSCs and enhance their proliferation and generation of ECM. Thus, our observations suggest that under normal conditions PSCs exert no effect on islet function, but in the hyperglycemic, inflammatory, and diabetic microenvironment they become activated and, as in the exocrine pancreas [8–10, 26–30], initiate intraislet fibrosis leading to deleterious effects on *β*-cell survival and function. In accordance with this, a recent study demonstrated PSCs localized around islets in young (6 weeks) GK rat pancreas, but not in age-matched Wistar rats [7], implicating PSCs at an early stage of the development of the diabetic phenotype.

Our* in vitro* studies confirm a direct interaction between PSCs and *β*-cells and implicate paracrine mediators as one mechanism for that interaction. Thus PSC-conditioned, cell-free medium had marked and consistent effects to reduce INS-1 cell viability and function. These observations are consistent with a recent study using the RIN5 *β*-cell line [20]. In that study, RIN5 cells were indirectly cocultured with PSCs, confirming a direct effect of PSC-derived soluble mediators on *β*-cell viability and function. In the current study we extended the* in vitro* observations to demonstrate that a high concentration of glucose aggravates the detrimental effects of pancreatic stellate cells on *β*-cell viability and function, consistent with the reduced glucose-induced insulin secretion in the GK rat recipients of intrapancreatic PSC transplantations.

Chronic exposure to elevated extracellular glucose is known to be a factor in the progressive loss of *β*-cell function in T2DM [36], often referred to as “glucotoxicity.” Our study confirmed the existence of glucotoxic effects on INS-1 cell survival and secretory function and also demonstrated that the deleterious effects of high glucose were additive to those of PSCs. One important mechanism of *β*-cell failure in T2DM is the development of ER stress as a response to an imbalance between the rate of protein synthesis and folding capacity of the endoplasmic reticulum in hypersecreting *β*-cells, eventually resulting in *β*-cell apoptosis [37, 38]. Our measurements of increased CHOP mRNA and protein levels in response to PSCs-CM and/or high glucose suggest that the detrimental effects of PSCs on *β*-cells may be associated with the activation of the ER stress response. Overall, our* in vitro* observations concur with our* in vivo* studies and suggest that the hyperglycemia and systemic inflammation associated with the onset of T2DM cause the activation of quiescent PSCs. The activated PSCs have deleterious effects on *β*-cell survival resulting in reduced *β*-cell mass and on *β*-cell function resulting in reduced insulin secretion with consequent impaired glucose tolerance. In this model, PSCs do not initiate the onset of T2DM but act to amplify the consequences of the hyperglycemic, inflammatory environment, enhancing the development of *β*-cell fibrosis and their ultimate functional failure. We therefore propose that PSCs offer a novel therapeutic target through which to slow or halt the progression of T2DM.
